# Experimental Analysis of Heat-Affected Zone (HAZ) in Laser Cutting of Sugar Palm Fiber Reinforced Unsaturated Polyester Composites

**DOI:** 10.3390/polym13050706

**Published:** 2021-02-26

**Authors:** Fathi Masoud, S. M. Sapuan, Mohd Khairol Anuar Mohd Ariffin, Y. Nukman, Emin Bayraktar

**Affiliations:** 1 Department of Mechanical and Manufacturing Engineering, University Putra Malaysia, Serdang 43400, Selangor, Malaysia; fathimasoud77@gmail.com (F.M.); khairol@upm.edu.my (M.K.A.M.A.); 2 Laboratory of Biocomposite Technology, Institute of Tropical Forestry and Forest Products (INTROP), University Putra Malaysia, 43400 UPM, Serdang 43400, Selangor, Malaysia; 3 Department of Mechanical Engineering, University of Malaya, Kuala Lumpur 50603, Wilayah Persekutuan Kuala Lumpur, Malaysia; nukman@um.edu.my; 4 School of Mechanical and Manufacturing Engineering, Supmeca-Paris, 3 rue Fernand Hainaut, 93400 Saint Ouen, France

**Keywords:** HAZ, laser cutting, natural fiber, composite, machining

## Abstract

In this paper, the influence of processing input parameters on the heat-affected zone (HAZ) of three different material thicknesses of sugar palm fiber reinforced unsaturated polyester (SPF-UPE) composites cut with a CO_2_ laser was investigated. Laser power, traverse speed, and gas pressure were selected as the most influential input parameters on the HAZ to optimize the HAZ response with fixing all of the other input parameters. Taguchi’s method was used to determine the levels of parameters that give the best response to the HAZ. The significance of input parameters was also determined by calculating the max–min variance of the average of the signal-to-noise ratio (S/N) ratio for each parameter. Analysis of variation (ANOVA) was used to determine each input parameter’s contribution to the influence on HAZ depth. The general results show that the minimum levels of laser power and the highest levels of traverse speed and gas pressure gave the optimum response to the HAZ. Gas pressure had the most significant effect on the HAZ, with contribution decreases as the material thickness increased, followed by the traverse speed with contribution increases with the increase in material thickness. Laser power came third, with a minimal contribution to the effect on the HAZ, and it did not show a clear relationship with the change in material thickness. By applying the optimum parameters, the desired HAZ depth could be obtained at relatively low values.

## 1. Introduction

Until recently, natural fibers have largely been regarded as waste, as they were not efficiently exploited. However, in recent times, natural fibers have been used in important industries, such as the manufacturing of composites, where they have been employed as materials to reinforce a wide range of matrices, whether synthetic polymers or polymers of natural origin [1,2,3,4]. The use of natural fibers has contributed in one way or another to the production of partially or completely environmentally friendly composite materials as alternative reinforcing materials for synthetic fibers, contributing to energy saving and reducing the use of environmentally unfriendly synthetic materials [5]. Natural fibers are also used in some applications that employ natural fibers to reinforce biopolymers that can degrade naturally; they contribute to the production of totally environmentally friendly materials that have become widespread, such as alternative packaging materials [6].

In this research, fibers from sugar palm (Arenga Pinnata Wurmb (MARR)) plants are investigated. In addition to sugar production, the extraction of sugar palm fibers used to reinforce different types of matrices has become an additional factor of importance for sugar palm trees, where the composites that employ sugar palm fibers as reinforcing materials have shown good mechanical and physical properties [7]. In the current research, a sugar palm fiber reinforced unsaturated polyester (SPF-UPE) composite cut by laser beam machining technology (LBM) was investigated to solve some of the defects caused by traditional cutting methods. It is known that composites are manufactured close to the near-net shape, but they still require final processes, such as drilling, cutting, trimming, and profiling [8,9,10,11,12]. The application of traditional cutting methods to cut composites has resulted in significant defects, such as poor surface quality, material damage, and dimensional instability due to the cutting forces associated with the traditional cutting process and due to the heterogeneous nature of the composites [13,14,15]. In order to avoid these defects, alternative, unconventional cutting techniques were considered [9]. Laser beam machining technology (LBM) is one of the most important methods employed to cut composites because it is a non-contact cutting method and it does not require much force to fix the workpiece, in addition to its high productivity and automation ability [16,17]. The mechanism of the laser cutting process involves raising the temperature of the material locally at the cutting zone, thereby causing the melting or evaporating of the material and removing it by the flowing gas associated with the cutting process [18,19]. This technique causes the emergence of the heat-affected zone (HAZ) which extends to the parts of the material adjacent to the cutting kerf, causing undesirable effects on material properties. The HAZ is mainly related to the input parameters of the laser cutting process [20,21].

Limited studies have been conducted with the aim of improving the HAZ of natural fiber-reinforced composites (NFRPs), which limits the possibility of generalizing the results of these studies to other types of NFRPs, especially as the results are strictly affected by the types of fibers and matrices in addition to the type of input parameters and their range [5]. Tamrin et al. [22] evaluated the HAZ as a response to input parameters, including laser power, traverse speed, stand-off- distance, and the number of beam passes. The study was conducted on a 0.4 mm material thickness of cotton fiber laminate reinforced phenolic resin composite cut by a low-power CO_2_ laser. The effect of traverse speed on the HAZ was the most significant, as HAZ depth is inversely proportional to traverse speed. Laser power came second in terms of the effect on the HAZ, as it increased with the increase in laser power. The lowest values of the HAZ were obtained at the minimum values of laser power and the maximum values of traverse speed with minimal contributions of the rest of the input parameters. Renu Tewari et al. [23] applied laser power and traverse speed as input parameters to evaluate the HAZ response of kenaf reinforced high-density polyethylene (HDPE) composite that was drilled with a low-power CO_2_ laser. The study covered three different material thicknesses (3, 6.7, and 10 mm). The study concluded that laser power and traverse speed are the influential parameters on the HAZ in the laser-drilled kenaf reinforced HDPE composite, as the desired values of the HAZ were obtained by applying the minimum values of laser power and the maximum values of traverse speed. Moreover, the change in material thickness considerably influenced the HAZ, as the highest depth of the HAZ was recorded at the minimum thickness (3 mm), while the lowest values of the HAZ were obtained in the case of the thicker material (10 mm). As previously mentioned, studies in this field are limited and do not cover a wide range of input parameter levels, nor can it be confirmed that they are generalizable to other types of natural fiber composites. The importance of this research lies in that it provides important data for the process of cutting SPF-UPE composites by laser beam machining technology in order to contribute to avoiding the defects resulting from traditional cutting methods. What distinguishes this study is its coverage of a wide range of the input laser power values, unlike previous studies that used a relatively low laser power. Furthermore, this study was carried out on three different thicknesses of the composite, unlike most previous studies, which focused on only one thickness, making their results difficult to generalize on other different thickness of the similar composites. It is also possible to benefit from the generalization of the results of this research when cutting other natural fiber reinforced polymers composites with laser technology, especially those materials that are similar to SPF-UPE in properties and chemical composition. Providing sufficient data for the process of cutting natural fiber composites contributes to the uptake of their use as possible alternatives to synthetic fibers composites and to employing natural fiber composites in various applications, such as aerospace, automobiles, marine applications, construction industries, sporting products, packaging, and electronic industries applications.

This research investigates and analyzes the effect of input parameters on the HAZ in the laser cutting of sugar palm fiber reinforced unsaturated polyester (SPF-UPE) composites with three different material thicknesses. Laser power, traverse speed, and assist gas pressure represent the variable input parameters in order to obtain the optimum response of the HAZ using the Taguchi statistical method to determine the optimum levels of the input parameters and their significance, and the study is enhanced by using the analysis of variance method (ANOVA) to determine the extent of each parameter’s contribution to the effect on the HAZ.

## 2. Materials and Methods

### 2.1. Composite Forming

The material used in the experiments was a sugar palm fiber reinforced unsaturated polyester (SPF-UPE) composite. Sugar palm fibers (SPFs) were washed with pure water, dried by hot air dryer, and then treated by 0.25 M/L NaOH with one-hour immersion, as this type of treatment showed good improvement in the mechanical properties of SPF [7,24]. After drying, the fibers were cut manually with lengths from 5 to 10 mm (average aspect ratio 25). Unsaturated polyester (UPE) was used as the matrices with fiber loading by 30%, as this fiber content produced good mechanical and physical properties [25,26,27]. Three types of molds with three different depths were used to produce three types of specimens of 2, 4, and 6 mm thicknesses with lengths of 210 mm and widths of 120 mm. The composite specimens were made using the hand lay-up technique. The molds were covered with a weight of 40 kg and were then disassembled; the specimens were extracted after 24 h.

### 2.2. Experimental Setup

The experiments were performed using a CO_2_ laser cutting machine (AMADA, FO 3015 M2 NT, Buena Park, CA, USA) with a CNC worktable and a maximum output power of 4000 Watt in 1500 Hz pulsed mode. The laser beam was focused using a 7.5” focal length lens, the focal point was on the top surface of the specimen, the nozzle diameter was 2 mm, the nozzle stand-off-distance was 1.5 mm, and air was used as the assist gas. Laser power, traverse speed, and assist gas pressure were chosen as the input parameters as they showed a considerable influence on the HAZ [5,28,29,30,31,32]. The other parameters were fixed, such as nozzle diameter focal length and nozzle stand-off distance. Three different material thicknesses of 2, 4, and 6 mm were studied with different input parameter levels.

### 2.3. Cutting Parameter Selection

**Figure 1:**
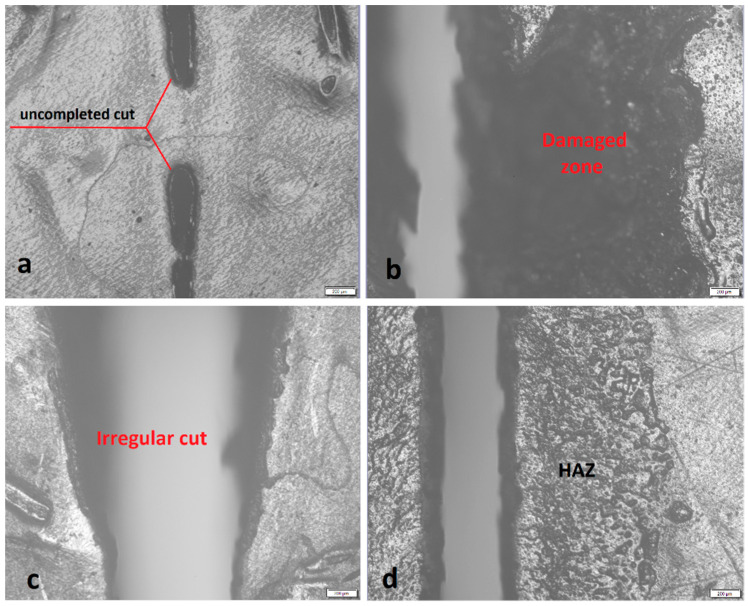
(**a**) Incomplete cut. (**b**) Observed damage at the cutting zone. (**c**) Irregular cutting kerf. (**d**) High depth of the heat-affected zone (HAZ).

Full thru cutting parameters (FTC) were determined for four different values of the laser power, namely, 100, 1000, 2000, and 3000 W, with fixed assist gas pressure at 2 bar, and then changing the traverse speed until its value at which the cut is full thru. This process was performed for every thickness. Thus, four groups of parameters for each thickness were obtained. Cases where no full cut occurred under any traverse speed value were excluded. Figure 1a shows an example of no full thru cut case. Three values were taken for each parameter in which the full thru cut was achieved. Then, nine cuts with the length of 60 mm were performed at different levels for the input parameters, according to L9 Taguchi array. Specimens that showed damage at the cutting zone and irregular kerf due to the large values of laser power, as shown in Figure 1b,c, respectively, were excluded. Additionally, the experiments that showed minimal productivity due to the low cutting speeds were excluded. The specimens that showed large values of the HAZ were also excluded, as illustrated in Figure 1d. 

The specimens with the lowest values of the HAZ were selected for study and optimization. In cases involving close HAZ values, priority was given to cases involving higher traverse speed. Table 1, Table 2 and Table 3 show the input parameter levels and full thru parameters founded for every thickness.

### 2.4. HAZ Measurement

**Figure 2:**
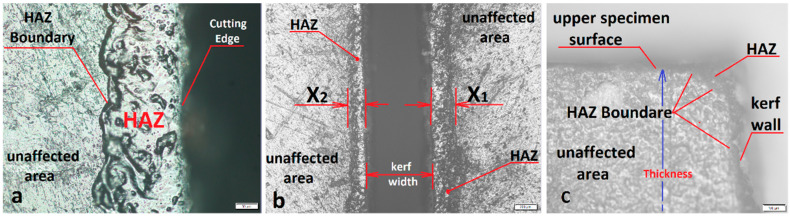
HAZ microscopic image. (**a**) HAZ boundary. (**b**) HAZ measurement method. (**c**) Optical image of the cross-section of the cut edge.

A reflected industrial microscope OLYMPUS BX51M system with Olympus Stream Essentials image analysis software (Olympus, Shinjuku, Tokyo, Japan) was used to measure HAZ depth. The HAZ and its boundary were observed clearly under the microscope as shown in Figure 2a. The HAZ was measured by taking ten readings along the cut (a reading every 5 mm) on both sides of the cut, as illustrated in Figure 2b, and then taking the average value. This process was conducted for the top and bottom of the specimen, but the upper surface was mostly considered because it involved the higher values of the HAZ in general. To analyze the HAZ at the cross-section, the samples were cut with a High-Speed Steel (HSS) saw and polished with abrasive paper to evaluate the HAZ depth at the material’s internal layers. The maximum HAZ values were recorded at the top surface of the material; meanwhile, the minimum HAZ depth was observed at the internal layers of the composite, as shown in Figure 2c.

### 2.5. Optimization Methods

The design of experiments (DOE) of the Taguchi method was used to investigate the effect of input parameters on the HAZ, where measured values of HAZ depth were analyzed using the signal-to-noise ratio (*S*/*N*) small-is-better calculation to determine the desired parameters that give the smallest HAZ depth and to identify a significant rank for every input parameter. The analysis of variance (ANOVA) method was adopted to determine the contribution of every input parameter to the HAZ property. All statistical calculations were generated by Minitab software (State College, PA, USA).

## 3. Results and Discussion

**Figure 3:**
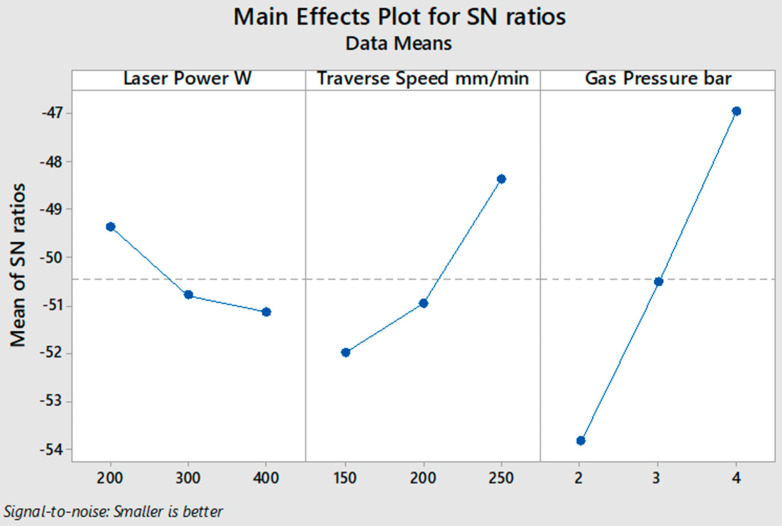
*S*/*N* ratio smaller-is-better average value of input cutting parameters of the 2 mm thick sugar palm fiber reinforced unsaturated polyester (SPF-UPE) composite.

Figure 3 shows the average values of the *S*/*N* ratio for all levels of the input parameters according to the calculated *S*/*N* ratio of the measured response of the HAZ, as shown in Table 4 for 2 mm specimen thickness. In these experimental conditions, the low values of laser power and high values of traverse speed and gas pressure gave the optimum desired response of the HAZ with an apparent variation in significance. The level of influence of input parameters is calculated in Table 5 by computing the max–min variation of the average of the *S*/*N* ratio, where gas pressure ranked first as the most influential factor on the HAZ, and traverse speed and laser power came second and third, respectively. According to the ANOVA results in Table 6, the input parameters contributed 72.56% gas pressure, 18.62% traverse speed, and 3.27% laser power. From Table 4 and Figure 3, it can be observed that experiment number 3 shows the lowest value of the HAZ. Thus, 200 W laser power, 250 traverse speed, and 4 bar gas pressure are the optimum values of the input parameters for cutting 2 mm thickness of the SPF-UPE composite plate using CO_2_ laser cutting technology based on the desired value of the HAZ response.

**Figure 4:**
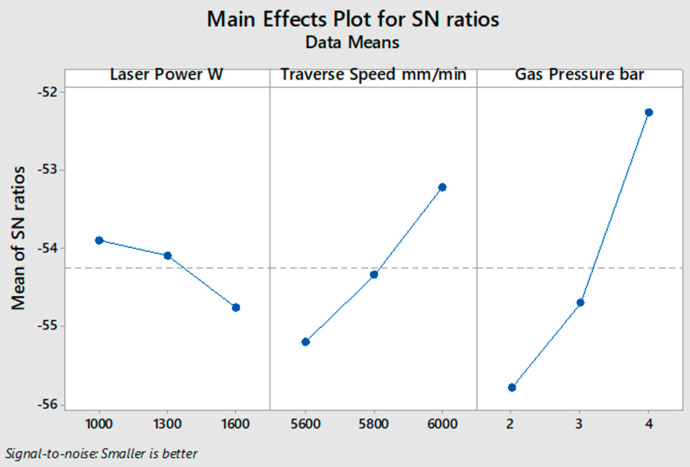
*S*/*N* ratio smaller-is-better average value of input cutting parameters of 4 mm thick SPF-UPE.

Figure 4 illustrates the average values of the *S*/*N* ratio for all levels of the input parameters according to the measured depth of the HAZ, as shown in Table 7 for 4 mm material thickness. In this experiment condition, the optimum desired response of the HAZ was obtained by applying a lower level of laser power and higher levels of traverse speed and gas pressure. The significance of the influence of input parameters is calculated in Table 8 by computing the max–min variation of the average of the S/N ratio, where gas pressure ranked first as the most influencing factor on the HAZ, and traverse speed and laser power ranked second and third, respectively. According to the ANOVA results in Table 9, there is a decrease in gas pressure contribution compared to that of the 2 mm material thickness case, with a minimal increase in traverse speed and laser power contributions. Input parameters in the case of 4 mm specimen thickness contributed 61.20% gas pressure, 20.14% traverse speed, and 3.88% laser power. From Table 7 and Figure 4, it can be observed that experiment number 3 shows the smallest HAZ depth. Thus, 1000 W laser power, 6000 mm/min traverse speed, and 4 bar gas pressure are the optimum values of the input parameters for cutting a 4 mm thick SPF-UPE composite plate using CO_2_ laser cutting technology.

**Figure 5:**
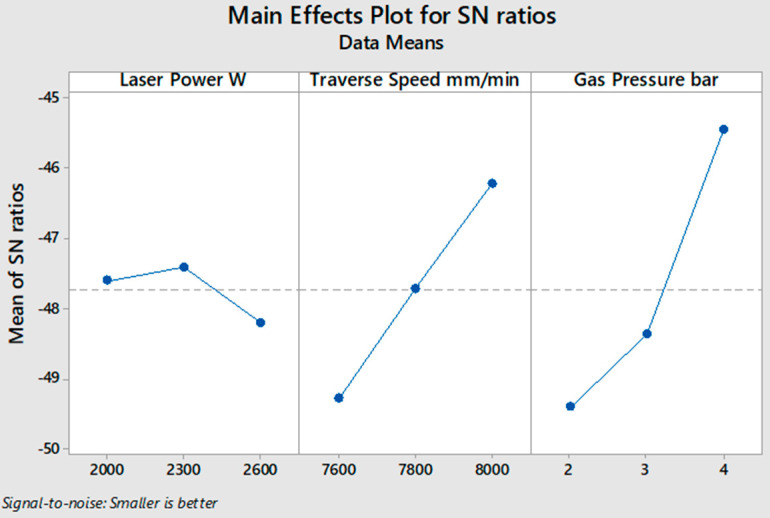
*S*/*N* ratio smaller-is-better average value of input cutting parameters of 6 mm thick SPF-UPE.

In the case of 6 mm material thickness, as shown in Table 10 and Figure 5, it is noted that the minimum value of the HAZ is obtained by applying the medium value of laser power, which is 2300 W, not at the minimum value, as in the cases of 2 and 4 mm specimen thicknesses. Moreover, as shown in Table 11, there is a significant increase in the contribution of traverse speed and a considerable decrease in gas pressure contribution, with a minimal decrease in the contribution of the laser power compared to the experiments with 2 and 4 mm of material thicknesses. According to the ANOVA results in Table 11, the input parameters in the case of 6 mm material thickness contributed 53.84% gas pressure, 33.26% traverse speed, and 1.58% laser power. The significance of the influence of input parameters is calculated in Table 12 by computing the max–min variation of the average values of the *S*/*N* ratio, as gas pressure came first as the most influential factor on the HAZ, and traverse speed and laser power ranked second and third, respectively. Based on *S*/*N* ratio analysis illustrated in Figure 5, the optimum parameters in cutting an SPF-PE specimen with a thickness of 6 mm using CO_2_ laser cutting technology are 2300 W laser power, 8000 mm traverse speed, and 4 bar assist gas pressure.

**Figure 6:**
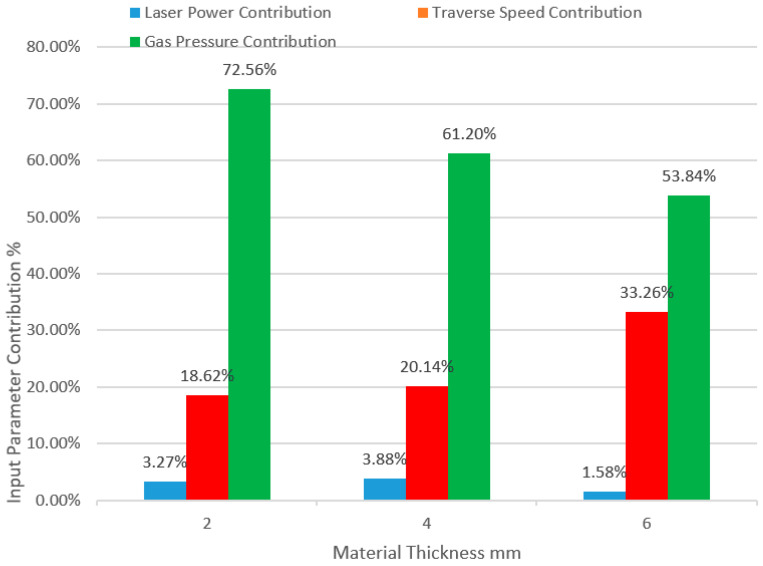
Input parameter contributions in HAZ of deferent material thicknesses.

**Figure 7:**
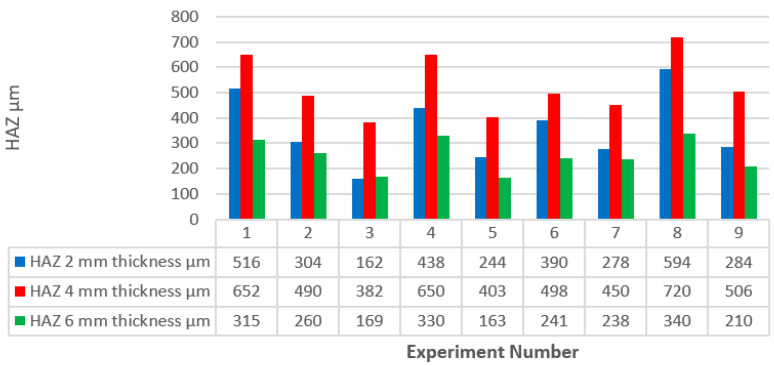
HAZ relative to experiment number and specimen thickness.

A comparison of the contributions of the input parameters for every thickness based on ANOVA results is illustrated in Figure 6. Gas pressure provides the highest contribution to the influence on the HAZ, and the minimum HAZ responses are recorded at the higher value of assist gas pressure, which can be explained as an increase in the rate of cooling associated with the increase in gas flow rate due to the increase in its pressure. This result was contrary to what Tamrin et al. [22], Renu Tewari et al. [23], Jose Mathew. [30], and Dalibor Petković [32] found, as traverse speed had the most considerable effect on the HAZ in their studies. Assist gas pressure contribution clearly decreases with the increase in material thickness, which may be a result of a reduction in the gas flow rate due to the effect of friction at the boundary layer along the passing area of the flow through the kerf, which increases with the thickness of the material. All experiments indicate a decrease in the propagation of the HAZ with an increase in the traverse speed, which may be due to the decrease in material exposure time to the laser produced heat, and this is consistent with what Tamrin et al. [22] found. Traverse speed rank came second in terms of effect on the HAZ, and its contribution increased with the thickness of the material. The minimum HAZ was recorded at minimum values of laser power in general, except for the case of 6 mm specimen thickness, where the optimum HAZ was recorded with a medium laser power value, which differs slightly from that recorded at the minimum value of laser power, and this is consistent with the results of the study conducted by Tewari et al. [23]. The laser power parameter contributed the least to the influence on the HAZ with no clear proportion with material thickness variation, and this is consistent with what Tamrin et al. [22] found. The lowest absolute values of the HAZ were recorded in the case of 2 mm material thickness followed by 6 mm material thickness, which also had the overall minimum average value, while the highest absolute and average values of the HAZ were recorded in the case of 4 mm material thickness. Figure 7 shows the value of the HAZ relative to the experiment number and specimen thickness.

## 4. Conclusions

Based on the results observed and discussed earlier, the conclusions can be summarized as follows:

- The CO_2_ laser is capable of cutting SPF-UPE composites. Optimum input cutting parameters can yield improved quality of the cut material by using LBM technology.
- Assist gas pressure has the greatest influence on the HAZ, followed by traverse speed and laser power, respectively, for all material thicknesses, noting that the contribution of gas pressure to the influence on the HAZ decreases with the increase in material thickness, while the contribution of traverse speed increases with the increase in material thickness. Moreover, it was observed that laser power minimally contributes to the influence on the HAZ, which did not show a clear relationship with the change in material thickness.
- The optimum responses of the HAZ were recorded by applying higher levels of traverse speed and gas pressure in all cases of material thickness. However, the optimum values of the HAZ were generally recorded at the lower levels of laser power in all cases of thicknesses, except for 6 mm material thickness, where the optimum HAZ was recorded at the medium level of laser power, but the value of the HAZ in this case did not differ much from that which was recorded at the minimum level of laser power.
- Based on DOE Taguchi and ANOVA analysis methods, the optimum values of input parameters and the contributions in the case of 2 mm material thickness were 200 W laser power with a contribution of 3.27% and 250 mm/min traverse speed with a contribution of 18.62%, while the optimum gas pressure was 4 bar with a contribution of 72.56%. In the case of 4 mm material thickness, the optimum laser power was 1000 W with a contribution of 3.88%, and 6000 mm/min traverse speed with a contribution of 20.14%, while the optimum gas pressure was also 4 bar with 61.20%. In the experiment of 6 mm specimen thickness, 2300 W laser power gave the lowest desired response of the HAZ with a minimal contribution of 1.58%, while 8000 mm/min was the optimum traverse speed with a contribution of 33.26%, and the optimum value of gas pressure was also 4 bar with a contribution of 53.84%.

## Figures and Tables

**Table 1:** Input parameters values for specimen thickness of 2 mm. Full thru cutting (FTC) parameters: 200 W laser power, 258 mm/min traverse speed, and 2 bar gas pressure.

Parameters	Level 1	Level 2	Level 3
Laser Power (W)	200	300	400
Traverse Speed (mm/min)	150	200	250
Gas Pressure (bar)	2	3	4

**Table 2:** Input parameters values for specimen thickness of 4 mm. FTC: 1000 W laser power, 6080 mm/min traverse speed, and 2 bar gas pressure.

Parameters	Level 1	Level 2	Level 3
Laser Power (W)	1000	1300	1600
Traverse Speed (mm/min)	5600	5800	6000
Gas Pressure (bar)	2	3	4

**Table 3:** Input parameters values for specimen thickness of 6 mm. FTC: 2000 W laser power, 8120 mm/min traverse speed, and 2 bar gas pressure.

Parameters	Level 1	Level 2	Level 3
Laser Power (W)	2000	2300	2600
Traverse Speed (mm/min)	7600	7800	8000
Gas Pressure (bar)	2	3	4

**Table 4:** L9 array of input parameter levels with measured HAZ and calculated signal-to-noise (*S*/*N*) ratio according to the Taguchi method for 2 mm specimen thickness.

Ex No:	Laser Power W	Traverse Speed mm/min	Gas Pressure bar	HAZ µm	S/N
1	200	150	2	516	−54.2530
2	200	200	3	304	−49.6575
3	200	250	4	162	−44.1903
4	300	150	3	438	−52.8295
5	300	200	4	244	−47.7478
6	300	250	2	390	−51.8213
7	400	150	4	278	−48.8809
8	400	200	2	594	−55.4757
9	400	250	3	284	−49.0664

**Table 5:** *S*/*N* ratio smaller-is-better response table of input cutting parameters of the 2 mm thick SPF-UPE.

Level	Laser Power W	Traverse Speed mm/min	Gas Pressure bar
1	−49.37	−51.99	−53.85
2	−50.80	−50.96	−50.52
3	−51.14	−48.36	−46.94
Delta	1.77	3.63	6.91
Rank	3	2	1

**Table 6:** ANOVA table for HAZ response of input cutting parameters of 2 mm thick SPF-UPE.

Source	DF	Seq SS	Contribution	Adj SS	Adj MS	F-Value	*p*-Value
Laser Power W	2	5048	3.27%	5048	2524	0.59	0.629
Traverse Speed mm/min	2	28,728	18.62%	28,728	14,364	3.36	0.229
Gas Pressure bar	2	111,944	72.56%	111,944	55,972	13.09	0.071
Error	2	8552	5.54%	8552	4276		
Total	8	154,272	100.00%				

**Table 7:** L9 array of input parameter levels with measured HAZ and calculated *S*/*N* ratio according to Taguchi methods for 4 mm specimen thickness.

Ex no:	Laser Power W	Traverse Speed mm/min	Gas Pressure bar	HAZ µm	S/N
1	1000	5600	2	652	−56.2850
2	1000	5800	3	490	−53.8039
3	1000	6000	4	382	−51.6413
4	1300	5600	3	650	−56.2583
5	1300	5800	4	403	−52.1061
6	1300	6000	2	498	−53.9446
7	1600	5600	4	450	−53.0643
8	1600	5800	2	720	−57.1466
9	1600	6000	3	506	−54.0830

**Table 8:** *S*/*N* ratio smaller-is-better response table of input cutting parameters of 4 mm thick SPF-UPE.

Level	Laser Power W	Traverse Speed mm/min	Gas Pressure bar
1	−53.91	−55.20	−55.79
2	−54.10	−54.35	−54.72
3	−54.76	−53.22	−52.27
Delta	0.85	1.98	3.52
Rank	3	2	1

**Table 9:** ANOVA table for the HAZ response of input cutting parameters of 4 mm thick SPF-UPE.

Source	DF	Seq SS	Contribution	Adj SS	Adj MS	F-Value	*p*-Value
Laser Power W	2	4384	3.88%	4384	2192	0.26	0.792
Traverse Speed mm/min	2	22,756	20.14%	22,756	11,378	1.36	0.423
Gas Pressure bar	2	69,147	61.20%	69,147	34,573	4.14	0.194
Error	2	16,690	14.77%	16,690	8345		
Total	8	112,977	100.00%				

**Table 10:** L9 array of input parameter levels with the measured HAZ and calculated *S*/*N* ratio according to Taguchi methods for 6 mm specimen thickness.

Ex No:	Laser Power W	Traverse Speed mm/min	Gas Pressure bar	HAZ µm	*S*/*N*
1	2000	7600	2	315	−49.9662
2	2000	7800	3	260	−48.2995
3	2000	8000	4	169	−44.5577
4	2300	7600	3	330	−50.3703
5	2300	7800	4	163	−44.2438
6	2300	8000	2	241	−47.6403
7	2600	7600	4	238	−47.5315
8	2600	7800	2	340	−50.6296
9	2600	8000	3	210	−46.4444

**Table 11:** ANOVA table for HAZ response of input cutting parameters of 6 mm thick SPF-UPE.

Source	DF	Seq SS	Contribution	Adj SS	Adj MS	F-Value	*p*-Value
Laser Power W	2	550.2	1.58%	550.2	275.1	0.14	0.877
Traverse Speed mm/min	2	11,557.6	33.26%	11,557.6	5778.8	2.94	0.254
Gas Pressure bar	2	18,710.2	53.84%	18,710.2	9355.1	4.76	0.174
Error	2	3933.6	11.32%	3933.6	1966.8		
Total	8	34,751.6	100.00%				

**Table 12:** *S*/*N* ratio smaller-is-better response table of input cutting parameters of 6 mm thick SPF-UPE.

Level	Laser Power W	Traverse Speed mm/min	Gas Pressure bar
1	−47.61	−49.29	−49.41
2	−47.42	−47.72	−48.37
3	−48.20	−46.21	−45.44
Delta	0.78	3.08	3.97
Rank	3	2	1

## Data Availability

The data presented in this study are available on request from the corresponding author.
